# Are the antidepressant effects of insulin-sensitizing medications related to improvements in metabolic markers?

**DOI:** 10.1038/s41398-022-02234-z

**Published:** 2022-11-08

**Authors:** Temi Toba-Oluboka, Kristýna Vochosková, Tomas Hajek

**Affiliations:** 1 Department of Psychiatry, Dalhousie University, Halifax, NS Canada; 2 National Institute of Mental Health, Klecany, Czech Republic; 3 Charles University, Third Faculty of Medicine, Prague, Czech Republic

**Keywords:** Depression, Prognostic markers

## Abstract

Insulin-sensitizing medications were originally used in psychiatric practice to treat weight gain and other metabolic side effects that accompany the use of mood stabilizers, antipsychotics, and some antidepressants. However, in recent studies these medications have been shown to cause improvement in depressive symptoms, creating a potential new indication outside of metabolic regulation. However, it is still unclear whether the antidepressant properties of these medications are associated with improvements in metabolic markers. We performed a systematic search of the literature following PRISMA guidelines of studies investigating antidepressant effects of insulin-sensitizing medications. We specifically focused on whether any improvements in depressive symptoms were connected to the improvement of metabolic dysfunction. Majority of the studies included in this review reported significant improvement in depressive symptoms following treatment with insulin-sensitizing medications. Nine out of the fifteen included studies assessed for a correlation between improvement in symptoms and changes in metabolic markers and only two of the nine studies found such association, with effect sizes ranging from R^2^ = 0.26–0.38. The metabolic variables, which correlated with improvements in depressive symptoms included oral glucose tolerance test, fasting plasma glucose and glycosylated hemoglobin following treatment with pioglitazone or metformin. The use of insulin-sensitizing medications has a clear positive impact on depressive symptoms. However, it seems that the symptom improvement may be unrelated to improvement in metabolic markers or weight. It is unclear which additional mechanisms play a role in the observed clinical improvement. Some alternative options include inflammatory, neuroinflammatory changes, improvements in cognitive functioning or brain structure. Future studies of insulin-sensitizing medications should measure metabolic markers and study the links between changes in metabolic markers and changes in depression. Additionally, it is important to use novel outcomes in these studies, such as changes in cognitive functioning and to investigate not only acute, but also prophylactic treatment effects.

## Introduction

Metabolic dysregulation, specifically metabolic syndrome (MetS) is common in bipolar disorders (BD) and major depressive disorder (MDD). Compared to the general population, people with severe mental illnesses (SMIs) have 1.6 times greater risk of MetS [1] and almost three times greater odds of having obesity [2]. The presence of MetS has implications for medical health and mortality, but it may also impact the clinical characteristics of the psychiatric disorder. Previous work has demonstrated that individuals with BD and MetS or its components have an increased severity of psychiatric symptoms, more lifetime episodes, more hospitalizations, shorter remissions, lower functioning, greater risk of disability, increased risk of suicide attempts, and impairments in cognitive functioning [3–12]. Additionally these individuals have poorer response to psychiatric medications [4, 13]. Consequently, it is important to test whether treatment of diabetes could improve some of these psychiatric outcomes. This is particularly relevant as the above-described psychiatric correlates of MetS are difficult to address with current medications.

Insulin-sensitizing medications were originally used in psychiatric practice to treat weight gain and other metabolic side effects that come with the use of mood stabilizers, antipsychotics and some antidepressants [14, 15]. Medications such as Metformin, Pioglitazone, Rosiglitazone, and SITAgliptin have proven efficacy in treating metabolic dysregulation and weight gain [15–18]. However, in recent studies, these medications have been shown to cause improvement in depressive symptoms, creating a potential new indication outside of metabolic regulation [19–21]. These studies have opened a new area of research that could potentially aid in the treatment of individuals with mood disorders. Studies and reviews have already suggested improvements of depressive symptoms with these insulin-sensitizing medications [22–24]. However, it is still unclear whether this improvement in depressive symptoms is associated with the improvement in metabolic markers. No review has specifically focused on this question. This is a key issue, especially as we generally do not understand the mechanisms through which psychiatric medications work. Linking improvements in psychiatric symptoms with specific changes in biochemical markers could inform testing of medications with new pharmacodynamic properties and it could also provide new techniques for monitoring or predicting psychiatric treatment outcomes. In addition, it is important to investigate the impact of diabetic treatments on outcomes, which are currently difficult to address, such as cognitive impairment, disability, and chronicity. If adjunctive treatment with antidiabetic medications can bring people closer to their premorbid functioning, then it is important to see what other factors may be playing a role in this improvement.

The present paper is a systematic review of studies using insulin-sensitizing medications to treat depressive symptoms. The specific question we wish to address is whether the improvement in depressive symptoms is connected to the improvement of metabolic dysfunction. In addition, we wanted to review how many of the studies also looked at additional psychiatric/cognitive outcomes, which have been associated with MetS or diabetes.

## Methods

In keeping with the PRISMA guidelines, we conducted a systematic PubMed search restricted to “human” participants and using the following search terms:(((“Depressive Disorder, Major”[Mesh] OR “Bipolar Disorder”[Mesh] or bipolar[tiab] OR depressi*[tiab])) AND (“Metformin”[Mesh] OR “Thiazolidinediones”[Mesh] OR metformin[tiab] OR thiazolidinedione*[tiab] OR pioglitazone*[tiab] OR rosiglitazone*[tiab] OR glitazone*[tiab])). We only included studies in the English language published up to January 2022. **(8,9,10)**. The bold numbers in parentheses are the PRISMA checklist items. The last search was performed on January 21st, 2022.

Using Covidence and following the PRISMA guidelines, we systematically screened and extracted studies that met the inclusion criteria as listed below **(1,2,3,4)**. A manual search was performed on the reference lists of included studies and systematic reviews **(5,11)**. No additional studies or data were sought by contacting authors, experts, manufacturers, or others **(6)**. Inclusion criteria was developed prior to the search as follows:

1. Individuals in a depressive episode with a primary diagnosis of depression or bipolar disorder
2. Prospective use of insulin-sensitizing medications as either adjunctive or monotherapy
3. Measurement of symptom improvement

We included randomized controlled trials and open-label studies fulfilling the above inclusion criteria. Considering the limited number of studies, including both types of studies seemed preferable to ensure a comprehensive review of the literature in comparison to solely focusing on RCT studies. We excluded conference abstracts as well as letters to the editor, systematic reviews, and meta-analyses.

**Fig. 1:**
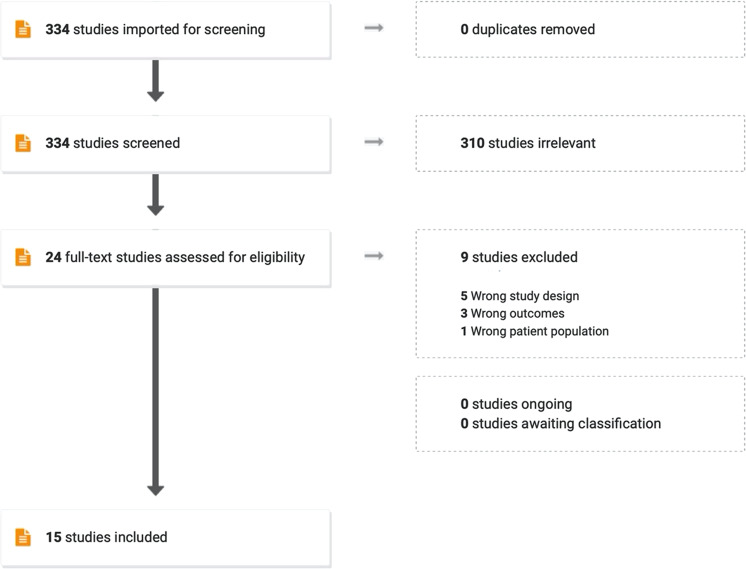
Schematic of screening process.

Two reviewers (TT-O, TH/KV) independently screened titles and abstracts to exclude articles that clearly did not fulfill the inclusion criteria. Out of 334 studies, 310 were excluded. Twenty-four potentially eligible studies were further assessed by retrieving full texts. Nine studies were excluded (5 for study design, 3 for outcomes, and 1 for participant population). Any disagreements were resolved by consensus or referral to a third reviewer (TH). A total number of 15 studies were included for further review **(14,15,16)** [22, 23, 25–37]. Please see Fig. 1 for a visual schematic of our screening process and Table 1 for a table of the included studies.

**Table 1:** Included studies.

Study	Type of study	Population	Medication	Length of study	Improved depressive symptoms	Improved metabolic markers	Metabolic comorbidities	Association between metabolic markers and mood symptoms
[25]	Double- blind RCT	80 MDD	Metformin or placebo	12 weeks	Yes	Not measured	No	Not assessed
[26]	Double- blind RCT	38 BD	Pioglitazone or placebo	8 weeks	No	Yes	Metabolic syndrome and insulin resistance	No
[27]	Open- label	86 PCOS with depressive symptoms	Metformin	3 months	Yes	Not measured	No	Not assessed
[28]	Open- label	44 PCOS with depressive and anxiety symptoms	Metformin	90 days	Yes	Yes	Insulin resistance	Not assessed
[29]	Single- blind RCT	58 with depression	Metformin or placebo	24 weeks	Yes	Yes	Type II diabetes	Yes
[30]	Open- label	118 PSD (post-stroke depression)	Pioglitazone and metformin^a^	12 weeks	Yes	Yes	Type II diabetes	Not assessed
[31]	Double- blind RCT	50 PCOS with MDD	Pioglitazone and metformin^a^	6 weeks	Yes	Yes	Insulin resistance	No
[32]	Open- label	23 MDD	Pioglitazone	12 weeks	Yes	Yes	Metabolic syndrome	No
[33]	Open- label	34 BD	Pioglitazone	8 weeks	Yes	Yes	Metabolic syndrome and insulin resistance	No
[34]	Double- blind RCT	37 MDD	Pioglitazone or placebo	12 weeks	Yes	Yes	Insulin resistance	Yes
[35]	Double- blind RCT	44 T2D with depressive symptoms	SITAgliptin or placebo	12 weeks	Yes	Yes	Type II diabetes	Not assessed
[22]	Open- label	10 unipolar 2 bipolar	Rosiglitazone	12 weeks	Yes	Yes	Insulin resistance	No
[36]	Double- blind RCT	104 MetS with depressive symptoms	Pioglitazone or placebo	24 weeks	Yes	Yes	Metabolic syndrome	No
[37]	Double- blind RCT	40 MDD	Pioglitazone or placebo	6 weeks	Yes	Not measured	No	Not assessed
[23]	Double- blind RCT	44 BD	Pioglitazone or placebo	6 w eeks	Yes	Not measured	No	Not assessed

^a^Metformin was used as a control group.

## Results

### Pioglitazone

Pioglitazone belongs to the thiazolidinediones (TZD) class and has been at the forefront of this research area, as one of the most used medications in studies assessing the potential antidepressant effect of antidiabetic medications. Two of the included studies were done by Kemp et al. which were both open-label studies [32, 33]. A third open-label study was done by Hu et al., [30]. The last six studies are double-blind randomized controlled trials (RCTs) that compared pioglitazone to placebo or metformin [23, 26, 31, 34, 36, 37].

### Open-label studies

Sample sizes for the included open-label studies ranged from 23 to 118 [30, 32, 33]. The studies broadly differed in their inclusion criteria. The first Kemp et al. study had a sample of individuals with either the presence of abdominal obesity or MetS, while their second study included individuals with either MetS or insulin resistance (IR) [32, 33]. The last open-label study included individuals with type 2 diabetes (T2D) [30].

In all of the open-label studies, there was an improvement in either depressive symptoms, anxiety or illness severity [30, 32, 33] after 8–12 weeks of treatment with pioglitazone, while one of these studies also reported significant improvement in functional disability [33].

In the first study, participants had a significant decrease in their fasting plasma glucose (FPG) at 12 weeks compared to baseline, as well as a significant decrease in fasting log insulin levels and IR [32]. However, the correlation between the change in IR and the change in depression severity was not significant [32]. Furthermore, reductions in depressive symptoms were comparable in those with versus without MetS [32]. In the second study, there was no association between high-sensitivity C-reactive protein (hsCRP), FPG, Insulin Sensitivity Index (ISI) or homeostasis model assessment of insulin resistance (HOMA-IR) and the change in depression severity [33]. Hu et al. reported a decrease in the fasting insulin levels (FINS) in the pioglitazone group but did not assess a connection between this improvement and depressive symptoms [30].

### RCT studies

Sample sizes of the included RCT studies ranged from 37 to 104 [23, 26, 31, 34, 36, 37]. Kashani et al. and Lin et al. conducted their respective studies in sample of individuals with IR [31, 34]. The Roohafza et al. study sample included individuals with IR while the Aftab et al. sample consisted of individuals with either MetS or IR [26, 36]. The Zeinoddini et al. and Sepanjnia et al. studies excluded individuals with history of T2D or MetS at baseline [23, 37].

Four of the six studies reported a significant improvement in the depressive symptoms of the pioglitazone group compared to the placebo or metformin group [23, 31, 36, 37]. In contrast, a single study reported a borderline significant improvement in symptoms in their placebo group in comparison to the pioglitazone group [26]. While one study did not find a significant difference in symptom improvement between their pioglitazone and placebo groups [34]. Additionally, all studies that measured metabolic markers reported improvements in them [26, 31, 34, 36]. Roohafza et al. found a change in the pioglitazone group’s HOMA-IR but they did not find a correlation between HOMA-IR and changes in depression scores [36]. Aftab et al. reported a significant decrease in HOMA-IR in the pioglitazone group; however, there was not a correlation reported between the metabolic marker and depressive symptoms [26]. Kashani et al. reported that the effect of pioglitazone on mood was independent of the drug insulin-sensitizing action as measured by HOMA-IR [31]. Specifically, there was no association between change in depressive symptoms and HOMA-IR change or HOMA-IR after treatment [31]. In addition, metformin had minimal effect on depressive symptoms (comparable to placebo) although it improved the HOMA-IR similar to pioglitazone [31].

Only one study reported an association between changes in metabolic markers and changes in depressive symptoms [34]. Within the pioglitazone group, change in HDRS-21 was positively correlated to change in oral glucose tolerance test (OGTT), R^2^ = 0.26 [34].

Two studies did not quantify changes in metabolic markers or assess the connection between the improvement in depressive symptoms and metabolic markers [23, 37]. Based on the registration, the Zeinoddini et al. study collected metabolic markers, but only mentioned that they did not differ between the groups [23].

### Rosiglitazone

Rosiglitazone is another insulin-sensitizing medication that belongs to the TZD class. There is only a single study investigating its antidepressant efficacy as an add-on treatment in 12 depressed individuals with IR [22]. Participants were given rosiglitazone as an add-on treatment to their antidepressant medications over a 12-week period [22]. This study reported a significant decline in participant’s depressive symptoms and CGI scores [22]. The Matsuda Index scores decreased, suggesting improvement of IR. There was no association between depression severity or change and metabolic markers or their change, other than an association between TG/HDL ratio and depression scores [22].

### Metformin

Metformin has been at the forefront of treating medication-related metabolic dysregulation and continues to be successful in doing so in practice. When it comes to studying its potential antidepressant efficacy, the literature is mostly centered on its uses in MDD. Two of the studies were open-label studies [27, 28]. The other two studies were blinded RCTs [25, 29].

### Open-label studies

Sample sizes ranged from 44 to 86 and both studies were completed in samples of individuals that did not have a known metabolic comorbidity [27, 28]. Both studies reported improvement in the depressive symptoms of people that were taking metformin for 3 months [27, 28]. The first study reported that after continuous use of metformin, there was significant reduction in participant’s bodyweight, BMI, waist to hip ratios, fasting concentrations of blood glucose, serum insulin levels [28]. The second open-label study did not assess metabolic related markers but did see improvements in participant’s emotional well-being, energy/fatigue [27]. Neither of the studies assessed the connection between metabolic markers and depressive symptom improvement [27, 28].

### RCT studies

Sample sizes ranged from 58 to 80. The first RCT had a sample of individuals without metabolic comorbidities, while the second study was in a sample of participants with T2D [25, 29].

Both RCT studies reported significant improvement in participant’s depressive symptoms in comparison to the placebo groups after 8 and 12 weeks of treatment with metformin [25, 29].

Abdallah et al. study did not assess metabolic markers [25]. The last RCT study reported a significant decrease of HbA1c levels after treatment with metformin [29]. Depressive symptoms positively correlated with HbA1c levels at the end of the study with an effect size of, R^2^ = 0.38 [29]. Additionally, metformin improved participant’s cognitive performance in verbal memory index, visual memory index, general memory index, attention, and concentration as well as delayed memory tasks [29]. These facets of cognitive functioning were negatively correlated with depression scores, with participants scoring higher on these tasks as their depression scores decreased [29]. Based on these findings, the authors suggested that antidepressant effects of metformin were related to stable blood glucose levels and improved cognitive functioning.

### SITAgliptin

To date, there has only been one study assessing SITAgliptin for treatment of depressive symptoms in individuals with T2D [35]. This study is a double-blind RCT with 44 participants with T2D and depressive symptoms [35]. In their analysis of depressive scores, the placebo group was favored on one measure but this finding was not statistically significant. However, on a second self-report measure, SITAgliptin was superior to placebo in alleviating depressive symptoms [35].

For metabolic markers, there was a moderate to large difference in HbA1c observed for the SITAgliptin group [35]. In contrast, SITAgliptin’s impact on HOMA-IR and fasting glucose were not statistically significant [35]. Moulton et al. also explored changes in inflammatory markers. HsCRP had a small to moderate change during the study, however this change was not statistically significant [35]. This study did not explore the connection between the change in depressive symptoms and changes in metabolic markers.

## Discussion

The majority of studies included in this review reported that treatment with insulin-sensitizing medications resulted in a significant improvement in participant’s depressive symptoms and in most RCTs this improvement was significant in comparison to placebo or metformin. Most of the studies also found improvements in metabolic markers. However, only 8 out of the 15 included studies assessed for association between improvement in psychiatric symptoms and improvement in metabolic markers and only two of these eight studies reported such association [29, 34]. Specifically, improvements in OGTT and HbA1c were associated with improvements in depressive symptoms during treatment with pioglitazone [34] or metformin [29]. Interestingly, both of these studies were RCTs, although one of them was single blinded. At the same time in the seven other RCTs, authors either did not report associations between metabolic changes and symptom improvements [23, 25, 35, 37] or found no such associations [26, 31, 36].

The fact that associations between symptoms and improvements in metabolic markers were found only in RCTs, but not in open-label observational studies is interesting. Perhaps there is a patient or prescriber bias, which makes it difficult to detect such associations, but it is not readily clear what such bias could be. However, the overall pattern even in RCTs is far from clear, as three other RCTs found no associations between improvements in metabolic and mood symptoms. Specifically, one RCT reported improvement in IR in absence of changes in depressive symptoms [31]. The largest RCT reported that changes of HOMA-IR scores in their pioglitazone group were not correlated with the changes in depression scores, even though both HOMA-IR and depression scores improved in this study [36]. The third study failed to demonstrate antidepressant efficacy of pioglitazone over placebo and in fact showed a trend favoring placebo. There were no statistically significant correlations between symptoms and inflammatory markers or insulin resistance (HOMA-IR) in this study, even though there was a significant decrease in HOMA-IR with pioglitazone compared to placebo [26].

Interestingly, whereas most studies reported improvements of depressive symptoms and metabolic markers during the treatment, six studies that assessed for association between depressive symptoms and metabolic markers, including the three RCTs, did not find one [22, 26, 31–33, 36]. One study even reported improvement in IR in absence of changes in depressive symptoms [31]. This interesting discrepancy generates many questions and could also motivate the design of future studies. There are potential methodological reasons for the preponderance of negative findings, including small sample sizes, broad inclusion criteria, broad diagnostic standards, resulting in patient and treatment heterogeneity with regards to both psychiatric and metabolic phenotypes. On the other hand, if the improvement in depressive symptoms was strongly related to improvement in metabolic markers, we would have seen a more consistent picture, even in the presence of some heterogeneity across studies. Alternatively, it is possible that the consistently reported improvement in depressive symptoms on insulin-sensitizing medications is related to other physiological or psychological processes such as anti-inflammatory properties or weight loss which may result in improvement in cognitive functioning [29, 38] or brain structure [39].

Mood disorders have been previously connected to neuroinflammation, demonstrated by an increase in cytokine levels, findings of peripheral markers of oxidative stress, glial pathology, blood-brain barrier dysfunction, and glutamate dysregulation [40]. Both clinical and preclinical findings suggest neuroinflammation as a key factor that interacts with the three known neurobiological pathways of major depressive disorder: dysregulation of the hypothalamo–pituitary–adrenal axis, depletion of brain monoamines, and alteration of neurogenesis in the dentate gyrus of the hippocampus [41]. Sustained neuroinflammation can lead to synaptic impairment, neuronal death and cause flare ups in multiple pathologies in the brain including mood disorders [42, 43]. Several preclinical or clinical studies have described an anti-inflammatory potential for antidiabetic agents acting either directly on inflammatory pathways, or indirectly by controlling hyperglycemia [44–46].

Another frequently described issue which is associated with obesity is systemic inflammation [47]. Medications, which specifically target inflammation may show some benefit in psychiatry [24, 48, 49]. However, one of the included studies did not find an association between improvement in depressive symptoms and inflammatory markers [35]. Our focus here was on antidiabetic medications, but considering their effects on inflammation, which may be relevant, future reviews should specifically focus on the broad category of anti-inflammatory medications.

Another mechanism through which insulin-sensitizing medications may improve mood symptoms might be the induction of weight loss. It is well established that obesity is a risk factor for mood disorders and vice versa [50–52]. In addition, obesity is consistently associated with cognitive impairment [53] and neurostructural alterations [54–57]. There is some evidence suggesting that weight loss may improve brain structure [58] which could also positively impact cognitive functions [59] and/or psychiatric symptoms [60]. Antidiabetic medications are often used in practice to assist in medication-related weight gain and other metabolic side effects that come with the use of psychiatric medications [15, 61]. In nine included studies, participants did not develop clinically significant weight gain or loss [22, 23, 25, 26, 31–35, 37]. Three studies did not evaluate changes in BMI [27, 30, 36]. Only a single study reported that metformin treatment significantly reduced bodyweight and BMI in 90 days follow-up [28]. All in all, as most studies did not report weight change in people whose depression improved, weight loss does not seem to be a key mediator of the improvement, but this still would require further research.

One key question in these studies is whether the positive effects of insulin-sensitizing medications occur only in people with IR or diabetes or whether these medications could help even individuals with mood disorders who do not suffer from MetS. A few studies looked at this [23, 25, 27, 37]. Two studies did not find differences in symptom improvement between people with and without MetS [32, 34]. This together with a lack of association between changes of insulin or glucose levels and depressive symptoms may suggest that IR or T2D are not a predictor of response to antidepressant properties of insulin-sensitizing medications. However, future studies should test for interaction between the presence/absence of T2D/IR and response to insulin-sensitizing medications.

An additional question is whether insulin-sensitizing medications may have stronger antidepressant properties in people with IR which may be reversible than in fully developed diabetes. In line with the staging models, one would expect a stronger effect in risk or precursor condition relative to the full-blown pathology. Interestingly, one of the 2 studies which showed association between improvement in metabolic and mood markers recruited individuals with diabetes [29]. So, there is some evidence that the antidepressant effects of these medications may manifest even in people with diabetes.

### Limitations

There are many limitations that impacted the present review paper. The diagnostic standards for depression were highly variable across all studies with some studies relying on self-report measures to classify depression versus a formal diagnosis [27, 35, 36]. Also, there was heterogeneity in using MetS as inclusion or exclusion criteria. Last but not least, it is important to report nonsignificant associations and analyze all outcomes, measures, as described in the study protocol/registration. Of note, one of the included RCTs has since been retracted due to concerns about the data presented [25]. It is also important to consider the severity of illness in these studies. More severe symptoms may result in greater improvements than milder symptoms. Three studies specifically looked at moderate to severely depressed patients and did see improvements [30, 34, 37]. However only one paper assessed both metabolic markers and symptoms and reported improvements in both variables [34].

Interestingly, only 8 out of the 15 included studies actually assessed for associations between changes in metabolic markers and changes in depressive symptoms. We generally do not know the mechanisms of action of psychiatric medications and do not have any biological correlates for monitoring of changes in depressive symptoms. Studies of medications with known pharmacodynamic properties provide ideal opportunities to obtain new insights into mechanisms of mood changes and for obtaining objective biological markers for monitoring of mood changes. It is unclear whether some of the studies did not measure metabolic markers or chose not to report their findings.

Obesity, diabetes or IR have been associated with greater number of psychiatric hospitalizations [7], lifetime depressive and manic episodes, greater risk of an affective recurrence, especially depressive [8], lifetime history of suicide attempt/s [9], as well as with greater rates of disability, chronicity [62, 63] lower response to psychiatric medications [4, 13], as well as with cognitive impairment [11] and brain alterations [39, 58]. Yet, few of these studies have looked at outcomes beyond symptoms, including general or cognitive functioning, risk of recurrence, stability of remission. One of the reviewed studies assessed overall functioning [27], and one included cognitive measures [29]. Considering the fact that obesity/diabetes/IR are associated with some of the currently intractable psychiatric outcomes, we need studies that go beyond symptom reduction and include additional outcomes, which are highly relevant for overall functioning, well-being of our patients.

### Future directions

Future studies of insulin-sensitizing medications should measure metabolic markers, and ideally use a standard panel of such markers, may be one including all features of metabolic syndrome. As the focus of these studies is on insulin sensitizing agents, they should include a measure of insulin resistance, which at the most basic form would require fasting samples of glucose and insulin. All such studies should also look at links between changes in metabolic markers and changes in depression and report the results regardless of statistical significance of the findings, so that they can be meta-analyzed along with the results of other studies. Registration of future studies would also help with similar reviews and meta-analyses and would guard against publication bias. Considering the absence of associations between improvements in metabolic and mood measures in most studies, future studies should include additional measures, such as for example inflammatory markers. Factors such as baseline presence of insulin resistance, diabetes or obesity could moderate the psychiatric response to these agents and should be carefully considered in the design and statistical analyses. It will also be important to use outcomes beyond just alleviation of depressive symptoms, i.e. improvements in cognitive, psychosocial functioning, brain structure and to investigate not only acute, but also prophylactic treatment effects, i.e. prevention of future episodes of depression or mania. Lastly, given the methodological heterogeneity of the included studies, it may be important that this area of research focuses more on producing RCTs. It is important to note that the studies that found a connection between metabolic markers and symptoms were both RCTs [29, 34].

## Conclusions

From the studies reviewed in the present paper, it is clear that the use of insulin-sensitizing medications have a clear positive impact on depressive symptoms. There is some evidence from RCTs that these improvements in psychiatric symptoms may be linked with improvements in glucose metabolism. At the same time, most studies, including RCTs found no association between improvement in depressive symptoms and improvement in metabolic markers or weight. In keeping with this, it seems that IR or diabetes are not pre-requisites for antidepressant response to insulin sensitizers. It is unclear which additional mechanisms play a role in the observed clinical improvement. Some alternative options include improvements in systemic or neuroinflammation, cognitive functioning or brain structure. The consistently reported improvements in psychiatric symptoms and metabolic markers, which do not appear to be associated with one another in most studies is an important impetus for future studies. This review serves as a reminder of just how important it is to continue to explore and investigate additional mechanisms that may play a role in psychiatric treatment response in order to continue to assist patients back to their previous states of functioning prior to illness.

## References

[1] Vancampfort D, Stubbs B, Mitchell AJ, De Hert M, Wampers M, Ward PB (2015). Risk of metabolic syndrome and its components in people with schizophrenia and related psychotic disorders, bipolar disorder and major depressive disorder: a systematic review and meta-analysis. World Psychiatry J World Psychiatr Assoc WPA.

[2] Afzal M, Siddiqi N, Ahmad B, Afsheen N, Aslam F, Ali A (2021). Prevalence of overweight and obesity in people with severe mental illness: systematic review and meta-analysis. Front Endocrinol.

[3] Calkin CV, Gardner DM, Ransom T, Alda M (2013). The relationship between bipolar disorder and type 2 diabetes: more than just co-morbid disorders. Ann Med.

[4] Calkin CV, Ruzickova M, Uher R, Hajek T, Slaney CM, Garnham JS (2015). Insulin resistance and outcome in bipolar disorder. Br J Psychiatry.

[5] Pan A, Lucas M, Sun Q, Van Dam RM, Franco OH, Manson JE (2010). Bidirectional association between depression and type 2 diabetes mellitus in women. Arch Intern Med.

[6] Silarova B, Giltay EJ, Dortland AVR, Van Rossum EFC, Hoencamp E, Penninx BWJH (2015). Metabolic syndrome in patients with bipolar disorder: comparison with major depressive disorder and non-psychiatric controls. J Psychosom Res.

[7] Cassidy F, Ahearn E, Carroll BJ (1999). Elevated frequency of diabetes mellitus in hospitalized manic-depressive patients. Am J Psychiatry.

[8] Fagiolini A, Kupfer DJ, Houck PR, Novick DM, Frank E (2003). Obesity as a correlate of outcome in patients with bipolar I disorder. Am J Psychiatry.

[9] Fagiolini A, Kupfer DJ, Rucci P, Scott JA, Novick DM, Frank E (2004). Suicide attempts and ideation in patients with bipolar I disorder. J Clin Psychiatry.

[10] Fagiolini A, Kupfer DJ, Masalehdan A, Scott JA, Houck PR, Frank E (2005). Functional impairment in the remission phase of bipolar disorder. Bipolar Disord.

[11] Salvi V, Di Salvo G, Korčáková J, Torriero S, Aragno E, Kolenič M (2020). Insulin resistance is associated with verbal memory impairment in bipolar disorders. J Affect Disord.

[12] Mora E, Portella MJ, Martinez-Alonso M, Teres M, Forcada I, Vieta E (2017). The impact of obesity on cognitive functioning in euthymic bipolar patients: a cross-sectional and longitudinal study. J Clin Psychiatry.

[13] Kemp DE, Gao K, Chan PK, Ganocy SJ, Findling RL, Calabrese JR (2010). Medical comorbidity in bipolar disorder: relationship between illnesses of the endocrine/metabolic system and treatment outcome. Bipolar Disord.

[14] McIntyre RS, McCann SM, Kennedy SH (2001). Antipsychotic metabolic effects: weight gain, diabetes mellitus, and lipid abnormalities. Can J Psychiatry.

[15] Newall H, Myles N, Ward PB, Samaras K, Shiers D, Curtis J (2012). Efficacy of metformin for prevention of weight gain in psychiatric populations: a review. Int Clin Psychopharmacol.

[16] Baptista T, Rangel N, Fernández V, Carrizo E, El Fakih Y, Uzcáteguiet E (2007). Metformin as an adjunctive treatment to control body weight and metabolic dysfunction during olanzapine administration: a multicentric, double-blind, placebo-controlled trial. Schizophr Res.

[17] Praharaj SK (2016). Metformin for lithium-induced weight gain: a case report. Clin Psychopharmacol Neurosci.

[18] Zheng W, Li XB, Tang YL, Xiang YQ, Wang CY, de Leon J (2015). Metformin for weight gain and metabolic abnormalities associated with antipsychotic treatment: meta-analysis of randomized placebo-controlled trials. J Clin Psychopharmacol.

[19] Colle R, De Larminat D, Rotenberg S, Hozer F, Hardy P, Verstuyft C (2017). PPAR-γ agonists for the treatment of major depression: a review. Pharmacopsychiatry.

[20] Kapadia R, Yi JH, Vemuganti R (2008). Mechanisms of anti-inflammatory and neuroprotective actions of PPAR-gamma agonists. Front Biosci J Virtual Libr.

[21] Yasmin S, Jayaprakash V (2017). Thiazolidinediones and PPAR orchestra as antidiabetic agents: from past to present. Eur J Med Chem.

[22] Rasgon NL, Kenna HA, Williams KE, Powers B, Wroolie T, Schatzberg AF (2010). Rosiglitazone add-on in treatment of depressed patients with insulin resistance: a pilot study. ScientificWorldJournal.

[23] Zeinoddini A, Sorayani M, Hassanzadeh E, Arbabi M, Farokhnia M, Salimi S (2015). Pioglitazone adjunctive therapy for depressive episode of bipolar disorder: a randomized, double‐blind, placebo‐controlled trial. Depress Anxiety.

[24] Jones BDM, Farooqui S, Kloiber S, Husain MO, Mulsant BH, Husain MI (2021). Targeting metabolic dysfunction for the treatment of mood disorders: review of the evidence. Life Basel Switz.

[25] Abdallah MS, Mosalam EM, Zidan AAA, Elattar KS, Zaki SA, Ramadan AN (2020). The antidiabetic metformin as an adjunct to antidepressants in patients with major depressive disorder: a proof-of-concept, randomized, double-blind, placebo-controlled trial. Neurotherapeutics.

[26] Aftab A, Kemp DE, Ganocy SJ, Schinagle M, Conroy C, Brownrigg B (2019). Double-blind, placebo-controlled trial of pioglitazone for bipolar depression. J Affect Disord.

[27] AlHussain F, AlRuthia Y, Al-Mandeel H, Bellahwal A, Alharbi F, Almogbel Y (2020). Metformin improves the depression symptoms of women with polycystic ovary syndrome in a lifestyle modification program. Patient Prefer Adherence.

[28] Erensoy H, Niafar M, Ghafarzadeh S, Aghamohammadzadeh N, Nader ND (2019). A pilot trial of metformin for insulin resistance and mood disturbances in adolescent and adult women with polycystic ovary syndrome. Gynecol Endocrinol J Int Soc Gynecol Endocrinol.

[29] Guo M, Mi J, Jiang QM, Xu JM, Tang YY, Tian G (2014). Metformin may produce antidepressant effects through improvement of cognitive function among depressed patients with diabetes mellitus. Clin Exp Pharm Physiol.

[30] Hu Y, Xing H, Dong X, Lu W, Xiao X, Gao L (2015). Pioglitazone is an effective treatment for patients with post-stroke depression combined with type 2 diabetes mellitus. Exp Ther Med.

[31] Kashani L, Omidvar T, Farazmand B, Modabbernia A, Ramzanzaeh F, Tehraninejad ES (2013). Does pioglitazone improve depression through insulin-sensitization? Results of a randomized double-blind metformin-controlled trial in patients with polycystic ovarian syndrome and comorbid depression. Psychoneuroendocrinology.

[32] Kemp DE, Ismail-Beigi F, Ganocy SJ, Conroy C, Gao K, Obral S (2012). Use of insulin sensitizers for the treatment of major depressive disorder: a pilot study of pioglitazone for major depression accompanied by abdominal obesity. J Affect Disord.

[33] Kemp DE, Schinagle M, Gao K, Conroy C, Ganocy SJ, Ismail-Beigi F (2014). PPAR-γ agonism as a modulator of mood: proof-of-concept for pioglitazone in bipolar depression. CNS Drugs.

[34] Lin KW, Wroolie TE, Robakis T, Rasgon NL (2015). Adjuvant pioglitazone for unremitted depression: Clinical correlates of treatment response. Psychiatry Res.

[35] Moulton CD, Rokakis AS, Pickup JC, Young AH, Stahl D, Ismail K (2021). SITAgliptin for depressive symptoms in type 2 diabetes: a feasibility randomized controlled trial. Psychosom Med.

[36] Roohafza H, Shokouh P, Sadeghi M, Alikhassy Z, Sarrafzadegan N (2014). A possible role for pioglitazone in the management of depressive symptoms in metabolic syndrome patients (EPICAMP Study): a double blind, randomized clinical trial. Int Sch Res Not.

[37] Sepanjnia K, Modabbernia A, Ashrafi M, Modabbernia MJ, Akhondzadeh S (2012). Pioglitazone adjunctive therapy for moderate-to-severe major depressive disorder: randomized double-blind placebo-controlled trial. Neuropsychopharmacology.

[38] Mansur RB, Ahmed J, Cha DS, Woldeyoannes HO, Subramaniapillai M, Lovshin J (2017). Liraglutide promotes improvements in objective measures of cognitive dysfunction in individuals with mood disorders: a pilot, open-label study. J Affect Disord.

[39] Mansur RB, Zugman A, Ahmed J, Cha DS, Subramaniapillai M, Lee Y (2017). Treatment with a GLP-1R agonist over four weeks promotes weight loss-moderated changes in frontal-striatal brain structures in individuals with mood disorders. Eur Neuropsychopharmacol J Eur Coll Neuropsychopharmacol.

[40] Najjar S, Pearlman DM, Alper K, Najjar A, Devinsky O (2013). Neuroinflammation and psychiatric illness. J Neuroinflammation.

[41] Troubat R, Barone P, Leman S, Desmidt T, Cressant A, Atanasova B (2021). Neuroinflammation and depression: a review. Eur J Neurosci.

[42] Dantzer R, O’connor JC, Freund GG, Johnson RW, Kelley KW (2008). From inflammation to sickness and depression: when the immune system subjugates the brain. Nat Rev Neurosci.

[43] García-Bueno B, Pérez-Nievas BG, Leza JC (2010). Is there a role for the nuclear receptor PPARγ in neuropsychiatric diseases?. Int J Neuropsychopharmacol.

[44] Mizoguchi M, Tahara N, Tahara A, Nitta Y, Kodama N, Oba T (2011). Pioglitazone attenuates atherosclerotic plaque inflammation in patients with impaired glucose tolerance or diabetes a prospective, randomized, comparator-controlled study using serial FDG PET/CT imaging study of carotid artery and ascending aorta. JACC Cardiovasc Imaging.

[45] Schöndorf T, Musholt PB, Hohberg C, Forst T, Lehmann U, Fuchs W (2011). The fixed combination of pioglitazone and metformin improves biomarkers of platelet function and chronic inflammation in type 2 diabetes patients: results from the PIOfix study. J Diabetes Sci Technol.

[46] Tahara A, Kurosaki E, Yokono M, Yamajuku D, Kihara R, Hayashzaki Y (2013). Effects of SGLT2 selective inhibitor ipragliflozin on hyperglycemia, hyperlipidemia, hepatic steatosis, oxidative stress, inflammation, and obesity in type 2 diabetic mice. Eur J Pharm.

[47] O’Rourke RW (2009). Inflammation in obesity-related diseases. Surgery.

[48] Rapaport MH, Nierenberg AA, Schettler PJ, Kinkead B, Cardoos A, Walker R (2016). Inflammation as a predictive biomarker for response to omega-3 fatty acids in major depressive disorder: a proof-of-concept study. Mol Psychiatry.

[49] McIntyre RS, Subramaniapillai M, Lee Y, Pan Z, Carmona NE, Shekotikhina M (2019). Efficacy of adjunctive infliximab vs placebo in the treatment of adults with bipolar I/II depression: a randomized clinical trial. JAMA Psychiatry.

[50] Allison DB, Newcomer JW, Dunn AL, Blumenthal JA, Fabricatore AN, Daumit GL (2009). Obesity among those with mental disorders: a National Institute of Mental Health meeting report. Am J Prev Med.

[51] Faith MS, Butryn M, Wadden TA, Fabricatore A, Nguyen AM, Heymsfield SB (2011). Evidence for prospective associations among depression and obesity in population-based studies. Obes Rev J Int Assoc Study Obes.

[52] McIntyre RS, Konarski JZ, Wilkins K, Soczynska JK, Kennedy SH (2006). Obesity in bipolar disorder and major depressive disorder: results from a national community health survey on mental health and well-being. Can J Psychiatry Rev Can Psychiatr.

[53] Elias MF, Elias PK, Sullivan LM, Wolf PA, D’Agostino RB (2003). Lower cognitive function in the presence of obesity and hypertension: the Framingham heart study. Int J Obes Relat Metab Disord J Int Assoc Study Obes.

[54] McWhinney SR, Brosch K, Calhoun VD, Crespo- Facorro B, Crossley NA, Dannlowski U, et al. Obesity and brain structure in schizophrenia—ENIGMA study in 3021 individuals. Mol Psychiatry. 2022. 10.1038/s41380-022-01616-5. 10.1038/s41380-022-01616-5 PMC9902274 35739320

[55] McWhinney SR, Abé C, Alda M, Benedetti F, Bøen E, Del Mar Bonnin C, et al. Diagnosis of bipolar disorders and body mass index predict clustering based on similarities in cortical thickness-ENIGMA study in 2436 individuals. Bipolar Disord. 2021. 10.1111/bdi.13172. 10.1111/bdi.13172 PMC9187778 34894200

[56] McWhinney S, Kolenic M, Franke K, Fialova M, Knytl P, Matejka M (2021). Obesity as a risk factor for accelerated brain ageing in first-episode psychosis—a longitudinal study. Schizophr Bull.

[57] McWhinney SR, Abé C, Alda M, Benedetti F, Bøen E, Del Mar Bonnin C (2021). Association between body mass index and subcortical brain volumes in bipolar disorders-ENIGMA study in 2735 individuals. Mol Psychiatry.

[58] Nota MHC, Vreeken D, Wiesmann M, Aarts EO, Hazebroek EJ, Kiliaan AJ (2020). Obesity affects brain structure and function- rescue by bariatric surgery. Neurosci Biobehav Rev.

[59] Veronese N, Facchini S, Stubbs B, Luchini C, Solmi M, Manzato E (2017). Weight loss is associated with improvements in cognitive function among overweight and obese people: a systematic review and meta-analysis. Neurosci Biobehav Rev.

[60] Ma J, Rosas LG, Lv N, Xiao L, Snowden MB, Venditti EM (2019). Effect of integrated behavioral weight loss treatment and problem-solving therapy on body mass index and depressive symptoms among patients with obesity and depression: the RAINBOW randomized clinical trial. JAMA.

[61] McIntyre RS, McCann SM, Kennedy SH (2001). Antipsychotic metabolic effects: weight gain, diabetes mellitus, and lipid abnormalities. Can J Psychiatry Rev Can Psychiatr.

[62] Hajek T, Hahn M, Slaney C, Garnham J, Green J, Růzicková M (2008). Rapid cycling bipolar disorders in primary and tertiary care treated patients. Bipolar Disord.

[63] Ruzickova M, Slaney C, Garnham J, Alda M (2003). Clinical features of bipolar disorder with and without comorbid diabetes mellitus. Can J Psychiatry Rev Can Psychiatr.

